# NaBH_4_ induces a high ratio of Ni^3+^/Ni^2+^ boosting OER activity of the NiFe LDH electrocatalyst†

**DOI:** 10.1039/d0ra06617f

**Published:** 2020-09-10

**Authors:** Yaqiong Wang, Shi Tao, He Lin, Shaobo Han, Wenhua Zhong, Yangshan Xie, Jue Hu, Shihe Yang

**Affiliations:** Guangdong Key Lab of Nano-Micro Materials Research, School of Chemical Biology, Biotechnology Shenzhen Graduate School, Peking University 518055 Shenzhen China chsyang@pku.edu.cn; Department of Chemistry, The Hong Kong University of Science and Technology Clear Water Bay, Kowloon Hong Kong China; School of Electronic and Information Engineering, Jiangsu Laboratory of Advanced Functional Materials, Changshu Institute of Technology Changshu 215500 China; Department of Materials Science and Engineering, Southern University of Science and Technology Shenzhen 518055 China; School of Physics, University of Electronic Science and Technology of China Chengdu 610054 China; Faculty of Science, Kunming University of Science and Technology Kunming 650093 China hujue@kust.edu.cn

## Abstract

Electrochemical water splitting is a promising way to produce hydrogen gas, but the sluggish kinetics of the oxygen evolution reaction (OER) extremely restrict the overall conversion efficiency of water splitting. Transition metal based LDHs (TM LDHs) are one of the most effective non-noble metal OER catalysts and have attracted wide interest, especially the nickel–iron LDH (NiFe LDH). The high valence Ni^3+^ species with a large coordination number play a vital role in OER catalysis. Herein, we report on a surprising discovery that reaction between NiFe LDH and NaBH_4_ with multi-hydrides induces vacancy formation around Fe^3+^ and enrichment in Ni^3+^, crucially activating the OER performance. The ratio of Ni^3+^/Ni^2+^ is found to be closely tied to the OER performance, nicely accounting for the leading role of Ni^3+^ ions in octahedral sites in electrocatalysis. Significantly, the NaBH_4_ treated NiFe LDH directly on nickel foam (NF), denoted as NaBH_4_–NiFe LDH@NF exhibited an outstanding OER performance with an overpotential of only 310 mV at 100 mA cm^−2^, and a Tafel slope of 47 mV dec^−1^. For the series of TM LDHs we studied with different metal combinations, the high valence metal ion is found to be positively related to OER performance.

## Abstract 2

Reaction between NiFe LDH and NaBH_4_ induces vacancies around Fe^3+^ and enrichment in Ni^3+^, crucially activating the OER catalyst leading to high performance.

## Graphical abstract

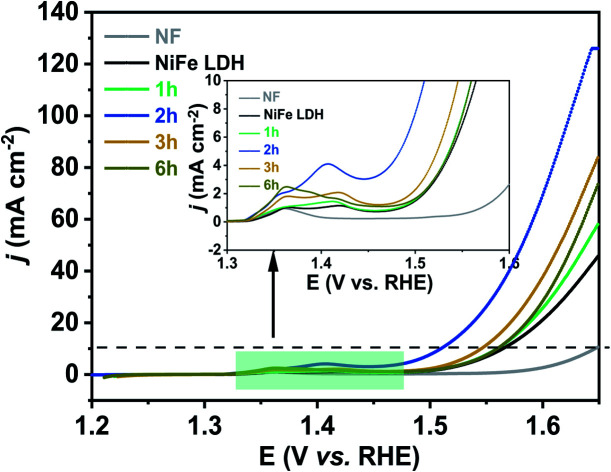

## Introduction

Hydrogen production by water splitting is one of the most promising ways to tackle the impending energy crisis and environment problems.^1–3^ In this process, the oxygen evolution reaction (OER) is the rate determining half reaction due to its four-electron transfer process leading to a high overpotential.^4–8^ Therefore, efficient yet low-cost oxygen evolution catalysts that could greatly accelerate the intrinsically slow kinetics and lower the unacceptable overpotential of OER are of key importance.^9^ In the past decades, great efforts have been made in exploring durable and efficient electrocatalysts.^10–14^ Unfortunately, noble metal-based materials, such as Pt, IrO_2_ and RuO_2_ are basically the most effective catalysts for water splitting.^15,16^ The expensive and scarcity of noble metals limit their practical application. Recently, effective transition metals based catalysts including transition metal oxides/hydroxides/oxyhydroxides^4,17–22^ have been well developed, among which, transition-metals based layered double hydroxides (TM LDH), especially the nickel–iron LDH (NiFe LDH), stand out among the highest performing ones.^23,24^

Extensive efforts have been devoted to understand the root cause behind the advanced catalytic performance of TM LDH. Many factors^25–30^ such as large specific surface area, good conductivity, and the synergistic effects between the transition metal ions have been proposed to account for the improved performance of TM LDH. Of particular importance and interest are to enhance the valence state metal ions such as Co^3+^ and Ni^3+^ which have been considered as the active sites^31–34^ for OER *via* inducing deprotonation of OOH species to produce oxygen due to its lower coordination number and higher adsorption energy of H_2_O.^35,36^ However, most of the works are focus on Co^2+^ and Ni^2+^ based catalysts.^37,38^ Therefore, it is extremely urgent to obtain electrocatalysts enriched in Ni^3+^ if one were to smarten the design and bring the highest performing NiFe LDH catalysts to the hydrogen production industry.^39,40^

New strategies are called for *in lieu* of the conventional way of tuning the chemical composition or structure to unravel the OER mechanism of NiFe LDH catalysts. Utilizing the widely used mild reductant NaBH_4_, NiFe LDH was able to be enriched in Ni^3+^, which turned out to be crucial in the OER catalysis.

## Experimental

### Synthesis of NiFe LDH

NiFe LDH nanosheets were synthesized by hydrothermal method. In a typical experiment, 0.145 ml of 1 M ferrous chloride (FeCl_3_) aqueous solution and 0.725 ml of 1 M nickel chloride (NiCl_2_) aqueous solution were mixed in the beaker with 70.8 ml DI water. Then 5.6 ml of 0.5 M urea aqueous solution and 2 ml of 0.01 M trisodium citrate (TSC) were added into the beaker under magnetic stirring. The mixed solution was then transferred to a 100 ml Teflon lined stainless steel autoclave and capped tightly for hydrothermal reaction in an oven at 150 °C for 24 h. After reaction, the powder was collected by repetitive centrifugation at 7500 rpm for 10 min and washed several times by de-ionized (DI) water and high purity ethanol, then dried at 50 °C in oven overnight. Similarly, in the same way, the nickel foam (NF) was immersed in Teflon containing 40 times diluted concentration of nickel nitrate and ferric nitrate to obtain the uniform NiFe LDH grown *in situ* on NF (NiFe LDH@NF).

### Synthesis of NaBH_4_–NiFe LDH

The NaBH_4_–NiFe LDH catalysts were synthesized by soaking the as-prepared NiFe LDH powder in 0.001 M NaBH_4_ solution at 40 °C for various times, respectively 1 h, 2 h, 3 h, and 6 h, then collected by repetitive centrifugation at 7500 rpm for 10 min, washed with DI water and dried in oven at 60 °C overnight. The reaction time was 2 h for NaBH_4_–NiFe LDH is not otherwise indicated. The NiFe LDH/NF was soaking in 0.001 M NaBH_4_ solution at 40 °C for 2 h to obtain NaBH_4_–NiFe LDH/NF.

### Synthesis of Ni(OH)_2_ for comparison

Ni(OH)_2_ was synthesized by hydrothermal method according to the same synthesis method with NiFe LDH. In a typical experiment, 0.87 ml of 1 M nickel chloride (NiCl_2_) aqueous solution were mixed in the beaker with 70.8 ml DI water. Then 5.6 ml of 0.5 M urea aqueous solution and 2 ml of 0.01 M TSC were added into the beaker under magnetic stirring. The mixed solution was then transferred to a 100 ml Teflon lined stainless steel autoclave and capped tightly for hydrothermal reaction in an oven at 150 °C for 24 h. After reaction, the powder was collected by repetitive centrifugation at 7500 rpm for 10 min and washed several times by DI water and high purity ethanol, then dried at 50 °C in oven overnight.

### Preparation of catalyst Ni foam electrodes

1 mg catalyst was dispersed in 0.25 ml ethanol uniformly by sonication for 2 hours, then mixed with 0.25 ml 4% PTFE containing 1 mg of catalyst. After sonication for 30 min, the catalyst ink was dropped onto a piece of Ni foam (1 cm × 1 cm) homogeneously and dried in oven at 60 °C for 15 min to obtain catalyst Ni foam electrode. Before dropped, the Ni foam was immersed in 1 M HCl solution for 10 min to remove the surface oxide, and then washed by DI and ethanol for several times and dried in oven at 60 °C.

### Electrochemical characterization

Electrochemical measurements were carried out in a standard three electrode system conducted by a CHI 660D electrochemistry workstation. The as-prepared catalyst Ni foam was used as the working electrode, platinum wire as the counter electrode and Hg/HgO electrode as the reference electrode. The reference was calibrated against and converted to reversible hydrogen electrode (RHE). All measurements were recorded in 1 M KOH. The cyclic voltammetry (CV) measurements were cycled at a scan rate of 10 mV s^−1^ for 20 times until a stable CV curve was achieved before measuring polarization curves of the catalysts. Linear sweep voltammetry (LSV) was carried out at 5 mV s^−1^ for the polarization curves and 1 mV s^−1^ for Tafel plots. LSV polarization curves were corrected with 95% *iR*-compensation.^41^ Chronopotentiometry (CP) was carried out under a constant current density of 10 mA cm^−2^, 20 mA cm^−2^, and 50 mA cm^−2^. Electrochemical impedance spectroscopy (EIS) analysis were conducted at 1.55 V *vs.* RHE at overpotential of 0.3 V at DC potential of 10 mV with the frequency ranging from 100 kHz to 0.1 Hz.

### Characterizations

The catalyst aqueous suspensions were drop-casted onto silicon wafer and the scanning electron microscopy (SEM) images were collected on a Zeiss Ultra 55 SEM at 5.0 kV and the energy dispersive X-ray spectroscopy (EDX) analysis was characterized by SEM Hitachi S-4800.

High Angle Annular Dark Field Scanning Transmission Electron Microscopy (HAADF-STEM) and STEM-EELS mapping were performed on a Double Cs-corrector FEI Titan Themis G2 60–300 microscope.

The crystal structure of samples was determined by X-ray diffraction (XRD, D8 Advance X-ray diffractometer) operated at 40 kV and 40 mA with a Cu Kα radiation (*λ* = 1.5405 Å) in the 2*θ* ranging from 10° to 80° with a step of 0.02°.

X-ray photoelectron spectroscopy (XPS) spectra were collected on Thermo ESCALAB 250XI (ThermoScientific). Spectra were analyzed using XPSPEAK software. The C1s peak for adventitious hydrocarbons at 284.8 eV was used for binding energy calibration.

XAS measurements were performed at 8-ID beamline of the National Synchrotron Light Source II (NSLS II) in the transmission mode at Brookhaven National Laboratory. The X-ray absorption near edge structure and extended X-ray absorption fine structure spectra were processed applying by the Athena software package. The AUTOBK code was used to normalize the absorption coefficient, and separate the EXAFS signal, *χ*(*k*), from the isolate atom-absorption background. The extracted EXAFS signal, *χ*(*k*), was weighted by *k*^3^ to emphasize the high-energy oscillations and then Fourier-transformed in a *k* range from 3.0 to 12.5 Å^−1^ to analyze the data in *R* space. Total scattering pair distribution function experiments were performed at beamline 28-ID-2 at NSLS-II of BNL using an amorphous silicon area detector (PerkinElmer) and an X-ray energy of 66.7 keV (*λ* = 0.185794 Å) to obtain data to large momentum transfer values. Data were integrated using the program Fit2D. PDFgetX3 was used to correct the data for background contributions, Compton scattering and detector effects, and to Fourier transform (*Q*_max_ = 23.5 Å) the data to generate *G*(*r*), the PDF.

### Calculation of faradaic efficiency (%) of O_2_

The amount of O_2_ evolution experimentally (*n*O_2_) was calculated to be 7.48 × 10^−5^ mol under constant oxidation current of 50 mA for 10 min. And the theoretically generated oxygen content 
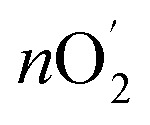
 was determined using Faraday's laws of electrolysis as follows: 
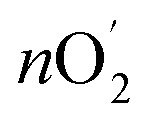
 = *Q*/4*F* = *I* × *t*/4*F* = 7.8 × 10^−5^ mol, where *Q* is measured charge, *I* is a constant oxidation current, *t* is the active time at the constant oxidation current, and *F* is Faraday constant, 96 485 C mol^−1^. Faradaic efficiency = 
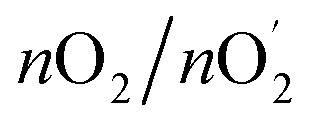
 = 4*Fn*O_2_/(*I* × *t*) = 95.9%.

## Results and discussion

**Fig. 1:**
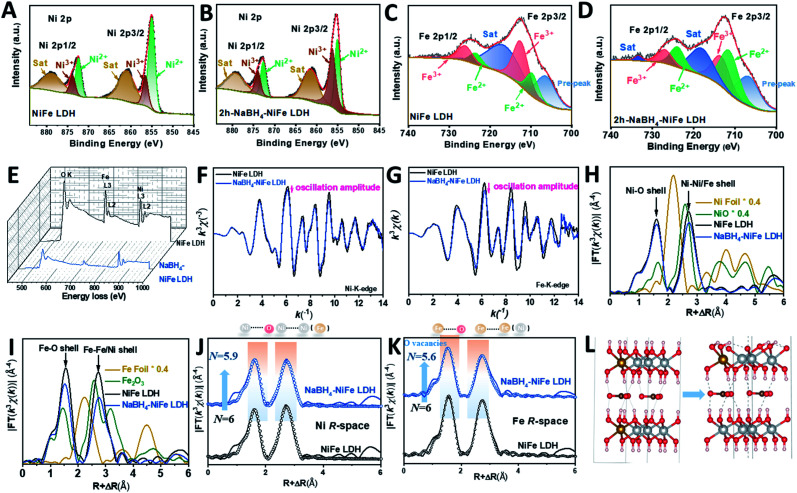
Experimental observation of the NaBH_4_–NiFe LDH enriched in Ni^3+^. High resolution XPS spectra of (A and C) NiFe LDH and (B and D) NaBH_4_–NiFe LDH in Ni 2p (A and B) and Fe 2p (C and D) regions, (E) EELS spectra of NiFe LDH (black curve) and NaBH_4_–NiFe LDH (blue curve), (F) Ni K-edge EXAFS oscillation functions *k*^3^*χ*(*k*), (G) Fe K-edge EXAFS oscillation functions *k*^3^*χ*(*k*), (H) detailed structural information shown in the *k*^3^-weighted FT spectra in *R*-space at the Ni K edge and (I) the Fe K edge, and (J) the fitted *R*-space plots at Ni K edge and (K) at Fe K edge for pristine NiFe LDH and NaBH_4_–NiFe LDH nanosheets, (L) local structural models of NiFe LDH (left), and NaBH_4_–NiFe LDH (right); Fe, Ni, O, H atoms are shown in orange, grey, red, and white, respectively.

Different from the previous reports in the literature,^42^ we used a dramatically reduced concentration of NaBH_4_ of 1 mM to obtain TM with high valence Ni (TM-HVN). This turned out to be crucial since the TM-HVN generated from the reduction by NaBH_4_ can easily undergo reductive elimination to evolve hydrogen (shown in Fig. S1†).^21,43–45^ The X-ray photoelectron spectroscopy (XPS) and electron energy loss spectroscopy (EELS) results were applied to examine the oxidation states of the TM ions before and after the NaBH_4_ treatment. From the high resolution XPS spectra in the Ni 2p region, Ni^3+^ and Ni^2+^ could be fitted at 856.2 eV and 855.1 eV, respectively, for both NiFe LDH (Fig. 1A) and NaBH_4_–NiFe LDH (Fig. 1B). Similar results were obtained in the Fe 2p region, with Fe^2+^ at 709.7 eV and Fe^3+^ at 712.5 eV, respectively (Fig. 1C and D).^46^ Most importantly, the ratio of Fe^2+^/Fe^3+^ and Ni^3+^/Ni^2+^ increased steeply from 0.40 and 0.39 for NiFe LDH to 1.49 and 1.32 for the NaBH_4_ treated sample denoted as NaBH_4_–NiFe LDH (Table S1†), respectively.

In addition, we collected EELS data and analyzed it using the Fourier-log method.^47^ Specifically, diverse intensity ratio of metal L_3_/L_2_ is correspondingly on behalf of metals in different valence states. For Fe L_3_/L_2_ in NiFe LDH, the value was 5.25, characteristic to Fe^3+^. And for Fe L_3_/L_2_ in NaBH_4_–NiFe LDH, the value decreased to 4.08 (Fig. 1E), depicting the reduced valence of Fe^3+^.^48^ And for Ni L_3_/L_2_, the values for NiFe LDH was estimated to be 3.16, corresponding to Ni^2+^. And the value was estimated to 4.08 in NaBH_4_–NiFe LDH, demonstrating the valence of Ni^2+^ increased to Ni^3+^. These results further confirm that the existed metal species in NaBH_4_–NiFe LDH are Fe^2+^ and Ni^3+^, which was in good agreement with the XPS results, and further confirmed the NaBH_4_ induced higher ratio of Ni^3+^/Ni^2+^. Meanwhile, the high-resolution O1s spectra of NiFe LDH (Fig. S2A†) revealed four distinct peaks attributed to the surface hydroxyl groups attached to metal–oxygen (531.5 eV), lattice oxygen (530.5 eV), under coordinated lattice oxygen related to oxygen vacancies (531.6 eV), and adsorbed water (532.8 eV).^49^ Indeed, a higher concentration of O vacancy was obtained from the deconvoluted O1s core-level spectra of NaBH_4_–NiFe LDH (Fig. S2B†) compared with NiFe LDH (Fig. S2A†), indicating the formation of O vacancies.

To further reveal the local chemical and electronic environment of the NiFe LDH and NaBH_4_–NiFe LDH, X-ray absorption near-edge structure (XANES) and extended X-ray absorption fine structure (EXAFS) were employed (Fig. 1F–K and S3†). As can be seen from two curves of XANES in Fig. S3A,† the *E*_0_ values embodied in the first inflection point on the edge of Ni K-edge in NaBH_4_–NiFe LDH had a higher shift compared to that of NiFe LDH. Since higher *E*_0_ corresponds to higher oxidation state,^50^ thus it can be concluded that the valence state of Ni species in NaBH_4_–NiFe LDH are higher than that in NiFe LDH, identical to the XPS result. On the contrary, as shown in Fig. S3B,† the *E*_0_ value of Fe K-edge in NaBH_4_–NiFe LDH had a lower shift compared to that of NiFe LDH, indicating a lower oxidation state of Fe ion in NaBH_4_–NiFe LDH. Furthermore, Fig. 1F showed that the Ni *K*-space spectra of the NaBH_4_–NiFe LDH exhibited fewer oscillations at high *k* values implying a subtle difference in the coordination environment of Ni atoms. The variation of oscillation is indicative of the change of coordination environment.^51^ Significantly, the variation of oscillations in Fe *K*-space between NaBH_4_–NiFe LDH and NiFe LDH, compared to that of Ni *K*-space was much more distinct, indicating a greater change in the coordination environment of the Fe atoms. The detail information about the Ni coordination was obtained from the corresponding *R* space plot in Fig. 1H and J, which exhibited the first shell (Ni–O) and second shell (Ni–Ni or Ni–Fe). The key information including the coordination number (*N*), the average distance (*R*) in each shell was shown in Table S2.† Compared with the Ni–O shell in NiFe LDH (*N* ≈ 6.0), the Ni–O shell for the NaBH_4_–NiFe LDH had barely unchanged *N* (5.9). Furthermore, the detail information about the Fe coordination was obtained from the corresponding *R* space plot (Fig. 1I and K, Table S2†). The Fe–O shell in NaBH_4_–NiFe LDH shown in Table S3† had a much lower *N* (5.6), compared with the Fe–O in NiFe LDH (6.0), indicating severe structural distortion caused by the abundance of oxygen vacancies in NaBH_4_–NiFe LDH. Meanwhile, the Debye–Waller factor (*σ*^2^) provides further evidence for severe structural distortion in the NaBH_4_–NiFe LDH. The larger Debye–Waller factor for Fe–O shell (0.0062 Å^2^) of NaBH_4_–NiFe LDH, compared with that of NiFe LDH (0.0047 Å^2^), suggested a severely distorted octahedral Fe–O environments after the NaBH_4_ treatment (Table S3†).^52,53^ Moreover, a mildly larger value for the Ni–O shell (0.0060 Å^2^) in NaBH_4_–NiFe LDH than that for NiFe LDH (0.0059 Å^2^) demonstrated a little distorted octahedral Ni–O environments (Table S2†).

On the basis of the results *vide supra*, a possible mechanism is proposed as shown in Fig. S4.† From Fig. S4A_1_†, the hydride from NaBH_4_ would grab one proton from hydroxide ligands of NiFe LDH, generating one molecule H_2_, along with the formation of a strong B–O σ bond (Fig. S4A_2_†). As the B–O bond has a strong tendency to form a B=O double bond due to the presence of empty p-orbital of B and lone pair of O, as well as the weak bond strength of B–H, hydride can grab another proton from NiFe LDH to form B=O double bond and release another molecule of H_2_ (Fig. S4A_3_†). Then, the BOH_2_^−^ dissociate from NiFe LDH to form oxygen vacancy near the iron (Fig. S4A_4_†). Since the presence of oxygen vacancy would inevitably lead to a reduced oxidation state of transition metals, and generally, transition metals with higher oxidation state are reduced firstly, it is reasonable for us to assume that Fe^3+^ was reduced. At the same time, the hydroxide bonded to nickel deprotonated by hydride in NaBH_4_, leads to the rise of oxidation state of nickel to maintain the neutrality. In essence, NaBH_4_ reacted with NiFe LDH to deprotonate the hydroxyl bonded to nickel and generate oxygen vacancy close to iron leading to the formation of Ni^3+^ and Fe^2+^ simultaneously. We found that the ratio of Ni^3+^/Ni^2+^ decreased after prolonged the time of NaBH_4_ treatment (longer than 2 h), whereas the ratio of Fe^2+^/Fe^3+^ remained at around 1.49 (Fig. S5 and Table S1†), indicating that 2 h was an optimal reaction time. The excess NaBH_4_ can coordinate to the vacant site of Fe^2+^, then the hydride can undergo reductive elimination with hydroxide ligand of NiFe LDH to generate water. Alternatively, an alpha-hydride migration occurs to transform oxy to hydroxyl, leading to the reduction of nickel. Fig. 1L showed the schematic process changing from NiFe LDH to NaBH_4_–NiFe LDH with O vacancies.

In order to further demonstrate the above-mentioned mechanism, the FT-IR spectra for LDH materials were shown in Fig. S6A.† The intense and broad peak at 3472 cm^−1^ was ascribed to O–H stretching vibration mode of water molecules which were intercalated within the interlaminar space. The band around 1630 cm^−1^ is related to the bending mode of those hydrogens bonded to water molecules. The sharp and strong band around 1360 cm^−1^ was responsible to the stretching mode of CO_3_^2−^ anions.^54^ Focused on the peak at around 750 cm^−1^ corresponding to Ni–OH, a slight shift to higher wavenumber was observed after NaBH_4_ treated, which was due to the formed double bond between nickel and oxygen. While the shoulder peak at 640 cm^−1^ related to residual Fe–OH groups has no obvious difference.^55^ Also, the Raman spectra (Fig. S6B†) showed a bond around 160 cm^−1^ which was associated with O–M–O bending modes, a minor shift to a higher shift was also in agreement with above mechanism shown in Fig. S4.†

**Fig. 2:**
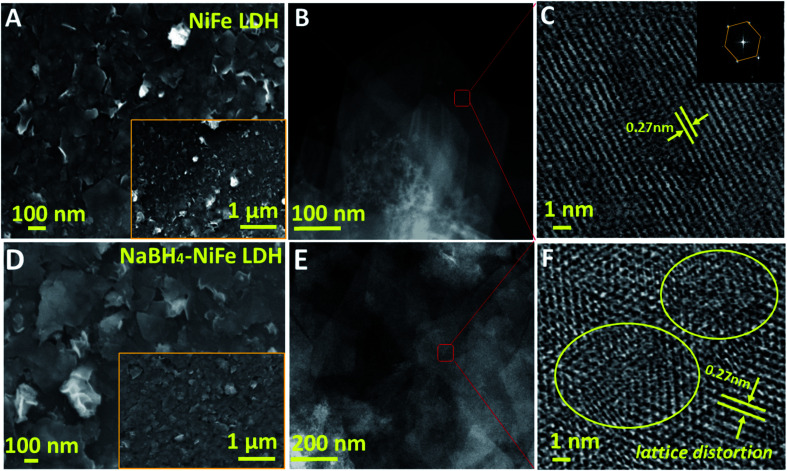
Morphology and structure characterizations. SEM (A and D), TEM (B and E) and HRTEM (C and F) images of NiFe LDH (A–C) and NaBH_4_–NiFe LDH (D–F). The yellow circles in F indicated lattice distortions.

Visually, the structure distortion was borne out from high resolution transmission electronic microscopy (HRTEM) images, wherein distortions could be found in NaBH_4_–NiFe LDH (Fig. 2F, yellow circles), instead of the continuous lattice fringes for NiFe LDH (Fig. 2C), suggesting the atomic structure modulation effect of NaBH_4_ treatment and the structural flexibility of the layer material. Nevertheless, the nanosheet structure and the crystalline phase of LDH were well retained after the NaBH_4_ treatment judging from the scanning electronic microscopy (SEM, Fig. 2A and D and S7†) and TEM (Fig. 2B and E) images and XRD (Fig. S8†) results.

**Fig. 3:**
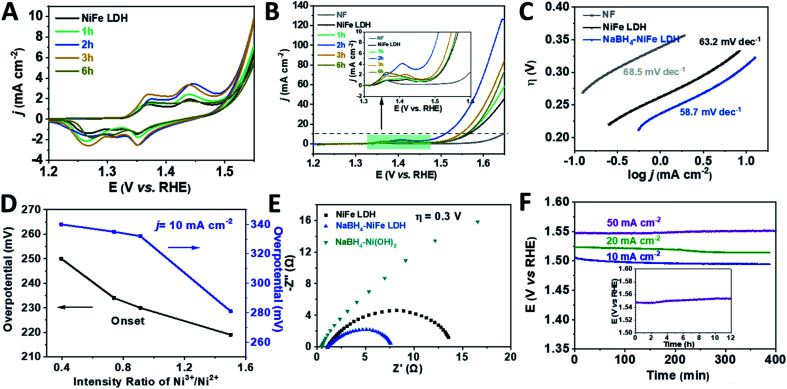
Electrocatalytic performance characterizations of the catalysts. (A) CV curves, (B) linear sweep voltammograms (LSV), (C) Tafel plots, (D) the overpotentials at onset and 10 mA cm^−2^ plotted against intensity ratio of Ni^3+^/Ni^2+^ after CV duration, (E) electrochemical impedance spectra (EIS) at the overpotential of 300 mV, (F) chronopotentiometry (CP) curves of NaBH_4_–NiFe LDH at 10 mA cm^−2^, 20 mA cm^−2^ and 50 mA cm^−2^; inset: long time operational stability testing with CP of NaBH_4_–NiFe LDH at the current density of 50 mA cm^−2^ for over 12 h.

Before the linear sweep voltammetry (LSV) measurements, cyclic voltammetry (CV) was carried out until the current was constant in the hope of identifying the catalytic active species of the catalysts. Furthermore, the ratio of the Ni^3+^/Ni^2+^ after the CV duration was analyzed by XPS and shown in Fig. S9 and Table S4.† Compared to the initial various NaBH_4_–NiFe LDHs, the ratio of Ni^3+^/Ni^2+^ was a bit higher after CV duration. From Fig. 3A, the NiFe LDH, NaBH_4_–NiFe LDH (1 h, 2 h, 3 h, and 6 h) showed anodic and cathodic peaks at ∼1.36 V (*vs.* RHE) and 1.43 V (*vs.* RHE) which are corresponding to simultaneous oxidation and reduction of Ni^2+^ to Ni^3+/4+^ system of Ni(OH)_2_ and NiOOH.^56–60^ Apparently, after NaBH_4_ treatment for 2 h, the charge of higher positive peak at 1.43 V (*vs.* RHE) was most and that of lower peak at ∼1.36 V (*vs.* RHE) was least among these LDHs. The positive shift of the redox couples resulted from the triggered high valence nickel species after NaBH_4_ treatment (the mechanism was shown in Fig. S3†).^57^ From the polarization curves (Fig. 3B) and Tafel plots (Fig. 3C), the NaBH_4_–NiFe LDH treated with NaBH_4_ for 2 h showed the lowest overpotential of 280 mV at 10 mA cm^−2^ and the smallest Tafel slope of 56 mV dec^−1^, indicating preferable catalytic performance on water oxidation. Moreover, the ratios of Ni^3+^/Ni^2+^ after CV duration calculated from the XPS data (Table S4†) were correlatively presented with the overpotentials of OER in Fig. 3D. One can see that the higher the ratio of Ni^3+^/Ni^2+^, the lower the OER onset potential, hence the better the catalytic performance. Therefore, it is reasonable to conclude that the OER performance is in proportion to the concentration of Ni^3+^, highlighting the importance of Ni^3+^ as the OER catalytic active centre, at least in part if Fe^3+^ would act as a synergistic partner. Understandably, a higher Ni^3+^ concentration would also mean a higher valence Ni species concentration with the rising potential, which may be the real oxidation state for assembling the O_2_ molecule and thus rounding off the OER process.

The electrochemical impedance spectroscopy (EIS) was performed to verify the above-mentioned reduction of charge transfer resistance due to the hydride treatment. As shown in Fig. 3E, the diameter of Nyquist semicircle of NaBH_4_–NiFe LDH is much smaller than that of NiFe LDH and NaBH_4_–Ni(OH)_2_, suggesting a much smaller charge transfer resistance of NaBH_4_–NiFe LDH due to the accelerated electron transfer through the distortion around metal Ni and Fe. On the other hand, the electrochemical active specific area (ECSA) of these catalysts were quite similar, with the estimated double layer capacitance (*C*_dl_) to be 2.8 mF, 2.2 mF, 3.3 mF, respectively (Fig. S10†), consistent with the retained microstructure of these catalysts.

The NaBH_4_–NiFe LDH catalyst also exhibited good durability for OER. As shown in Fig. 3F, after the duration of around 12 hours at current density of 50 mA cm^−2^, the anodic potential required to be kept well. The faradaic efficiency (FE) was calculated to be ∼96%, by comparing the amount of evolved H_2_/O_2_ (Fig. S11–S14†) with the consumed electricity, which comparable to the best reported transition metal based OER catalysts.

**Fig. 4:**
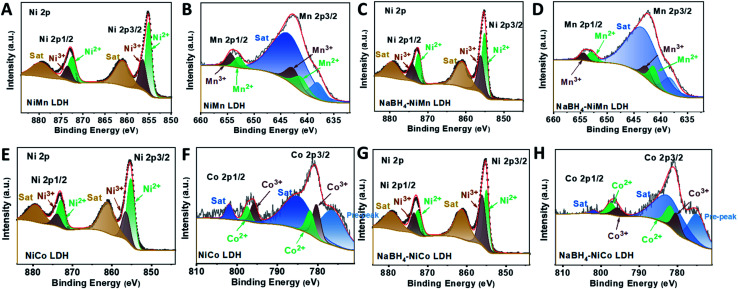
High resolution XPS spectra of Ni2p for (A) NiMn LDH and (C) NaBH_4_–NiMn LDH; Mn2p for (B) NiMn LDH and (D) NaBH_4_–NiMn LDH; Ni2p for (E) NiCo LDH and (G) NaBH_4_–NiCo LDH; Co2p for (F) NiCo LDH and (H) NaBH_4_–NiCo LDH.

To access the generality of the ligand engineering, NiMn, NiCo LDHs were also treated with NaBH_4_ by the same procedure. Both the Ni^3+^ and Ni^2+^ could be deconvoluted from the high resolution XPS spectra in the Ni region for both NiMn (Fig. 4A) and NaBH_4_–NiMn LDH (Fig. 4C). And the Mn 2p core line split into Mn^3+^ (642.7 eV and 653.9 eV)^61,62^ and Mn^2+^ (641.2 eV and 652.9 eV),^63^ respectively (Fig. 4B and D). The molar ratio of Ni^3+^/Ni^2+^ was increased from 0.5 to 1.01 and Mn^2+^/Mn^3+^ from 0.79 to 1.53 (Table S4†) after NaBH_4_ treatment. Similar phenomenon has been also observed in NiCo LDH by NaBH_4_ treatment (Fig. 4E–H). The estimated atomic ratios of Co^2+^/Co^3+^ and Ni^3+^/Ni^2+^ were increased from 0.76 to 1.42 and 0.45 to 1.20, respectively, after the NaBH_4_ treatment (Table S5†). The LSVs of all the LDHs and NaBH_4_-LDHs were presented in Fig. S15 and S16†. The abroad peak around 1.38 V corresponding to the pre-oxidation of nickel species^64^ shifted positively. And the overpotential at 10 mA cm^−2^ was both decreased during OER process for NiMn and NiCo LDHs. Hence, for the series of TM LDHs, after NaBH_4_ treatment, the relatively low valence metal ion was slightly increased and is found to be positively related to OER performance.

Although there is a shape increase of OER activity after NaBH_4_ treatment, the as prepared NaBH_4_–NiFe LDH which are glued to the nickel foam by PTFE can't afford a larger current density, hindering its practical application. Herein, we grew the NaBH_4_–NiFe LDH directly on nickel foam (NF), denoted as NaBH_4_–NiFe LDH@NF, which exhibited an overpotential of only 310 mV at 100 mA cm^−2^, and a Tafel slope of 47 mV dec^−1^ (Fig. S17†), outperforming most of the work shown in Table S6.† It should be noted the current density of the obtained NaBH_4_–NiFe LDH@NF can reach up to 800 mA cm^−2^ merely with an overpotential of 410 mV, suggesting its potential for industrial applications.

## Conclusions

We demonstrate for the first time a simple yet robust way to investigate muti-hydride of NaBH_4_ treatment induced vacancy and enrichment in high valence transition metal ion generation in TM LDH related to OER performance. Excitingly, the ratio of Ni^3+^/Ni^2+^ is found to be closely tied to the OER performance, nicely accounting for the leading role of Ni^3+^ ions in octahedral site in electrocatalysis. More importantly, this facile strategy is generalized to TM LDHs other than the NiFe LDH. Overall, this work sheds light on the catalytic active centre of TM LDH and enlightens the design and activation of transition metals based electrocatalysts for highly efficient water splitting and beyond.

## Conflicts of interest

There are no conflicts to declare.

## Supplementary Material

RA-010-D0RA06617F-s001
