# Supplementary material for: NaBH4 induces a high ratio of Ni3+/Ni2+ boosting OER activity of the NiFe LDH electrocatalyst
Source: RSC Adv. 2020 Sep 10;10(55):33475–82. doi: 10.1039/d0ra06617f (PMC9056658; doi:10.1039/d0ra06617f)
Supplement: RA-010-D0RA06617F-s001 [file RA-010-D0RA06617F-s001.pdf]

Electronic Supplementary Material (ESI) for RSC Advances.
This journal is © The Royal Society of Chemistry 2020

Supplementary Information

$NaBH_4$ Induces High Ratio of Ni^{3+}/Ni^{2+} Boosting OER Activity of the NiFe LDH Electrocatalyst†

Yaqiong Wang[a]‡, Shi Tao[c]‡, He Lin[b], Shaobo Han[d,e], Wenhua Zhong[a], Yangshan Xie[a], Jue Hu[f]* & Shihe Yang[a,b]*

[a]Guangdong Key Lab of Nano-Micro Materials Research, School of Chemical Biology and Biotechnology Shenzhen Graduate School, Peking University, 518055 Shenzhen, China. [b]Department of Chemistry, The Hong Kong University of Science and Technology, Clear Water Bay, Kowloon, Hong Kong, China. [c]School of Electronic and Engineering, Jiangsu Laboratory of Advanced Functional Materials, Changshu Institute of Technology, Changshu 215500, China. [d]Department of Materials Science and Engineering, Southern University of Science and Technology, Shenzhen, 518055, China. [e]School of Physics, University of Electronic Science and Technology of China, Chengdu 610054, China.[f]Faculty of Science, Kunming University of Science and Technology, Kunming, 650093, China.

‡ *these authors contribute equally to this work.* *E-mail addresses: chsyang@pku.edu.cn, hujue@kust.edu.cn

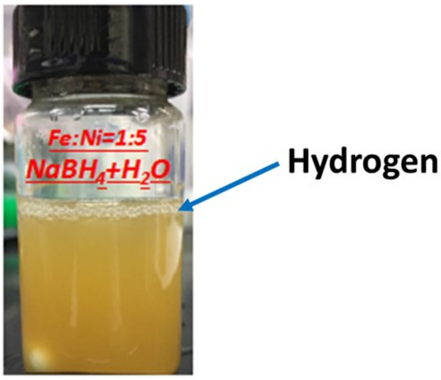

Fig. S1. The phenomenon of NiFe LDH activated by $NaBH_4$ treatment in aqueous solution.

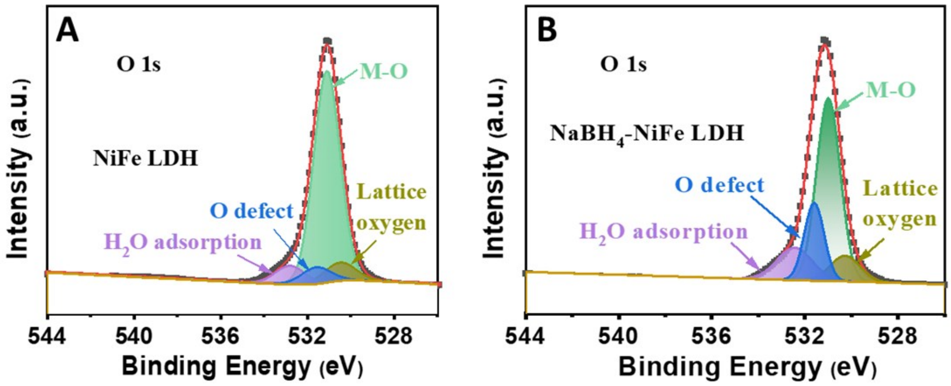

Fig. S2. High resolution XPS spectra of O1s for (A) NiFe LDH and (B) $NaBH_4$-NiFe LDH.

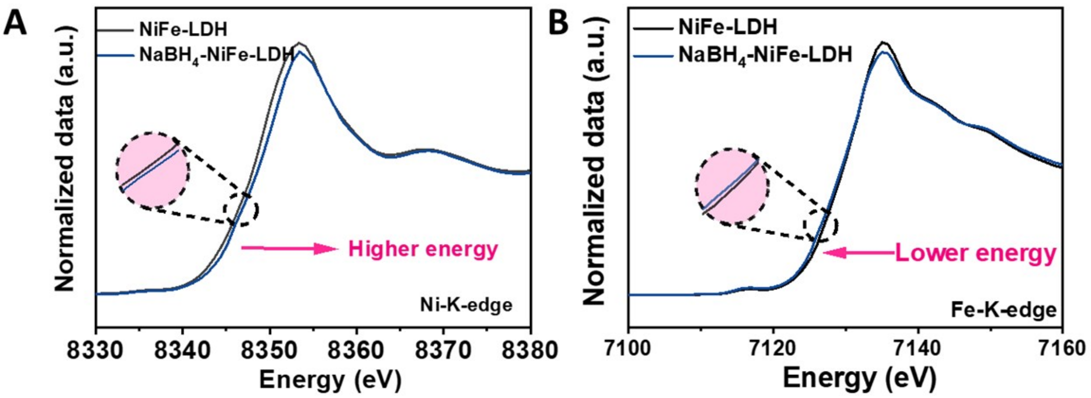

Fig. S3. (A) Ni K-edge XANES spectra, (B) Fe K-edge XANES data, the insets in A and B show magnified view of the rectangles.

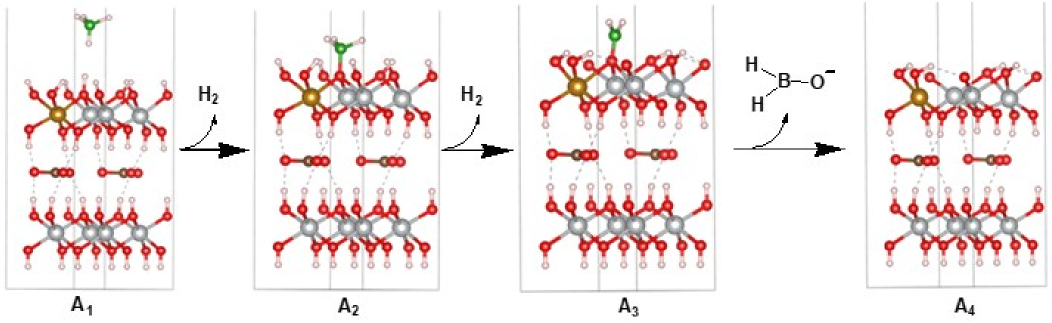

Fig. S4. Schematic illustration of the $NaBH_4$-triggered electron transfer activation process of NiFe LDH via $NaBH_4$ treatment: atomistic models.

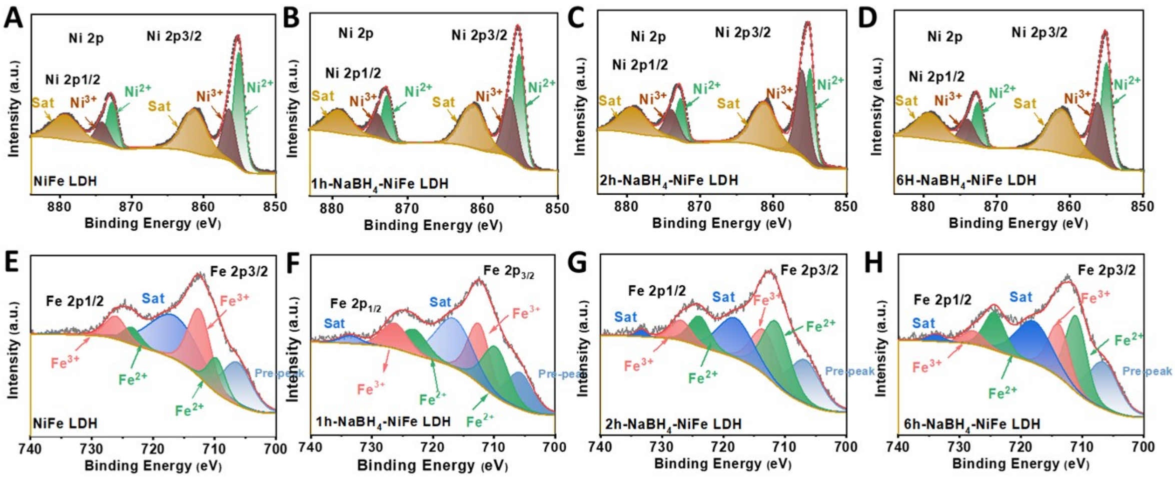

Fig. S5. Deconvoluted XPS spectra of NiFe LDH and NiFe LDH treated with $NaBH_4$ for 1, 2, and 6 hours in (A-D) Ni 2p spin-orbital and (E-H) Fe 2p spin-orbital.

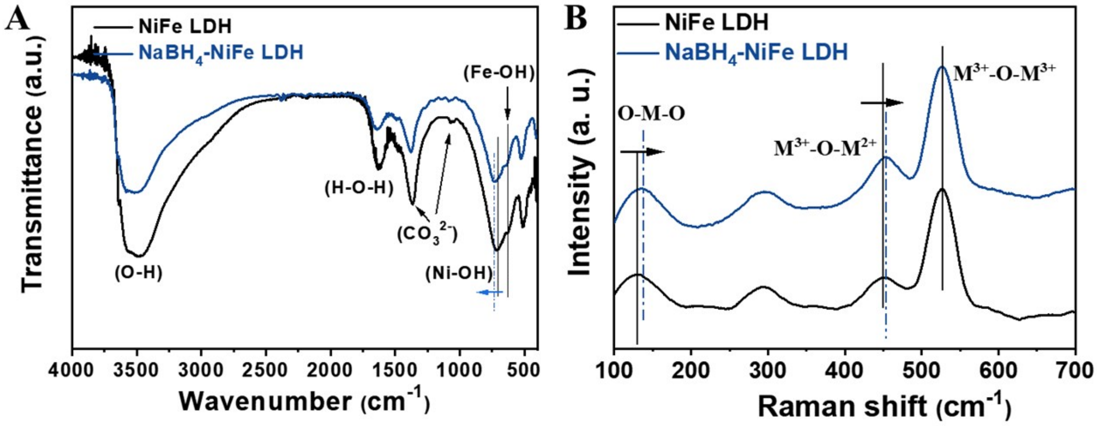

Fig. S6. FT-IR (A) and Raman (B) spectra of NiFe LDH and $NaBH_4$-NiFe LDH powders.

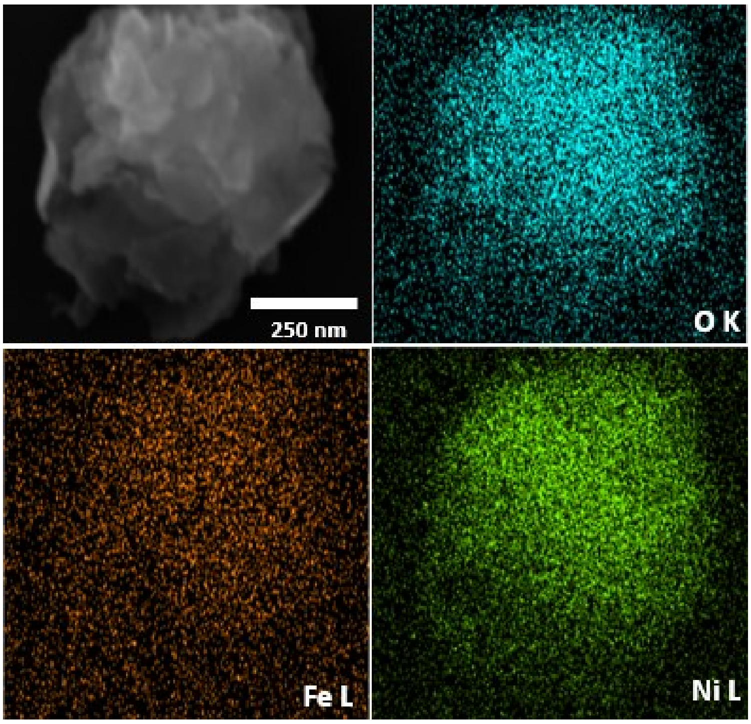

Fig. S7. Elemental mapping of $NaBH_4$-NiFe LDH.

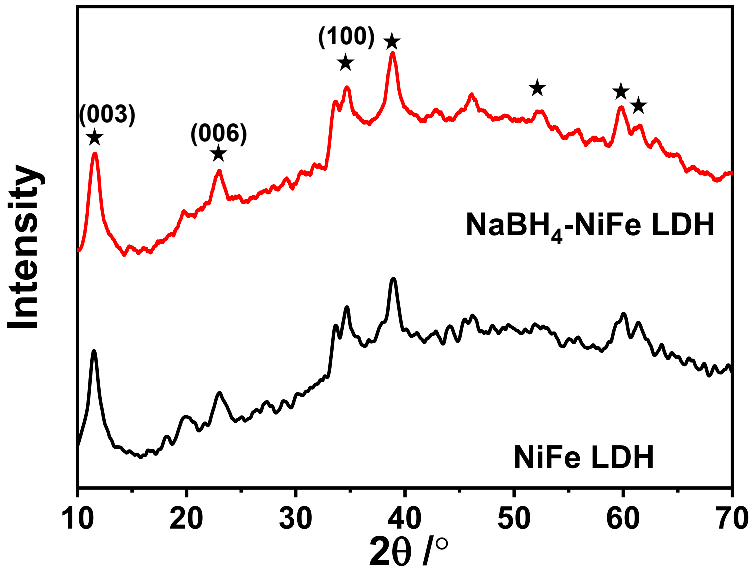

Fig. S8. XRD patterns of NiFe LDH (black curve) and $NaBH_4$-NiFe LDH (red curve).

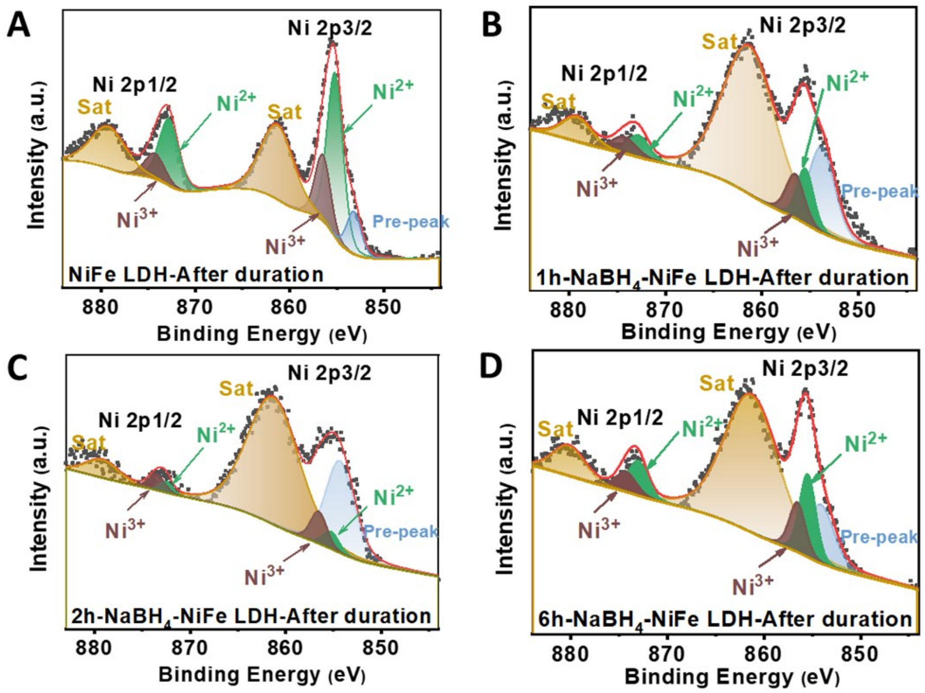

Fig. S9. Deconvoluted XPS spectra of NiFe LDH and $NaBH_4$-NiFe LDH for 1, 2, and 6 hours after CV duration in (A-D) Ni 2p spin-orbital.

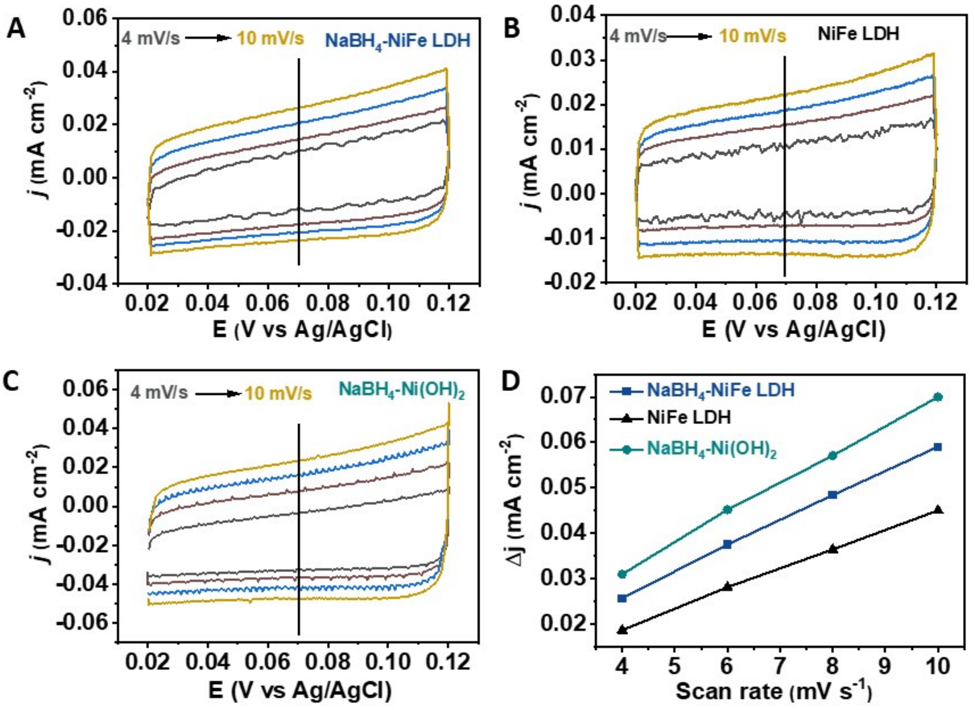

Fig. S10. The CV curves of **(A)** $NaBH_4$-NiFe LDH, **(B)** initial NiFe LDH and **(C)** $NaBH_4$-$Ni(OH)_2$ nanosheets with various scan rates, and **(D)** the capacitive current density differences plotted against scan rates.

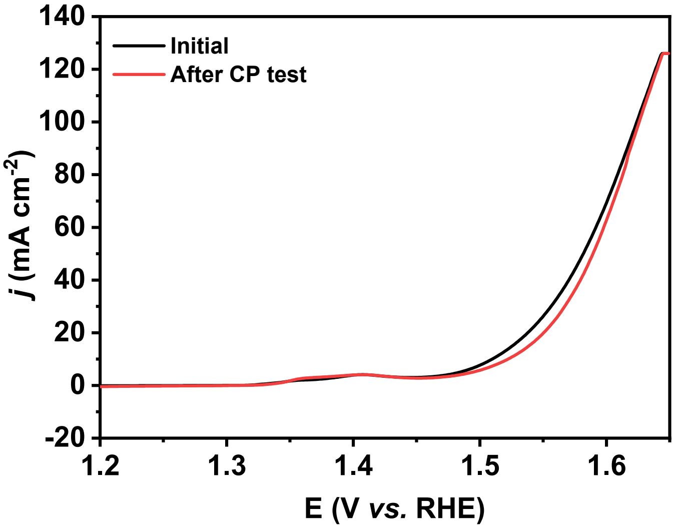

Fig. S11. LSV curves of initial $NaBH_4$-NiFe LDH nanosheets (black curve) and that after the CP testing at 50 mA cm^{-2} for 12 h (red curve).

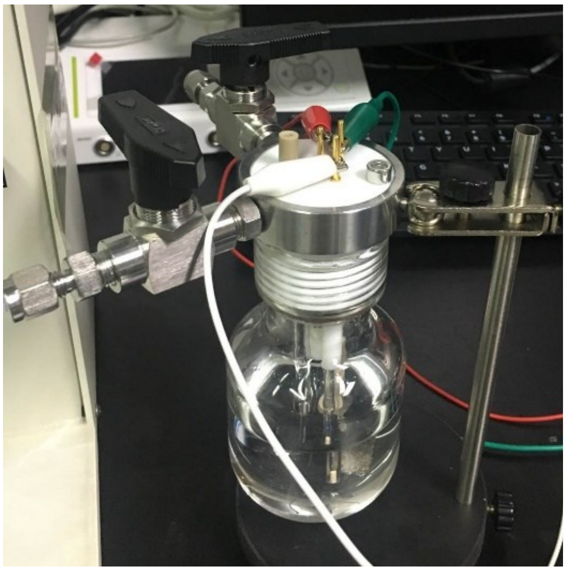

Fig. S12. The schematic diagram of OER equipment for FE measurement.

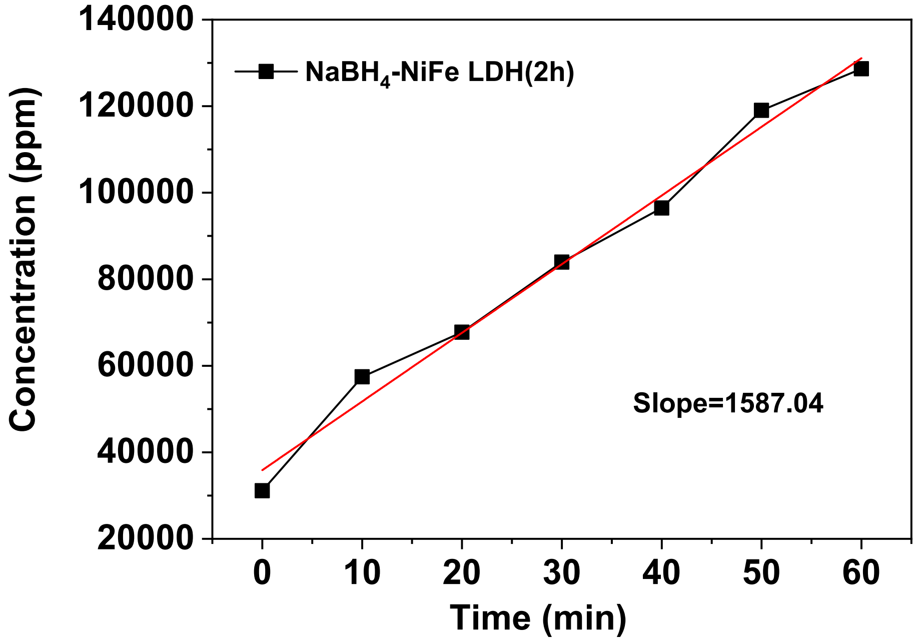

Fig. S13. The obtained concentration of O_2 from the GC analysis on $NaBH_4$-NiFe LDH (2h) under a constant oxidation current of 50 mA conducted by chrompotentiometry (CP) plotted against time injections. The volume of the sample injection is 1ml gaseous product.

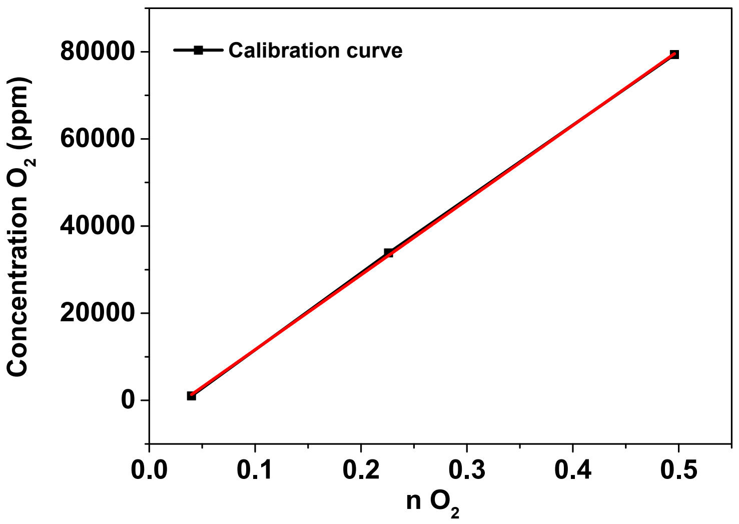

Fig. S14. The calibration curve prepared by injecting three calibrating gases with known concentrations of H_2 and O_2 into GC. The volume of the injection is 1 ml.

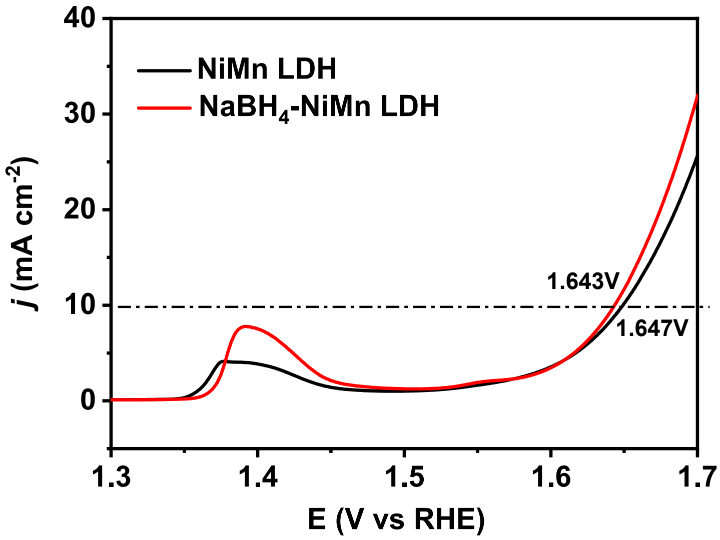

Fig. S15. Linear sweep voltammetry (LSV) curves of the NiMn and $NaBH_4$-NiMn LDHs

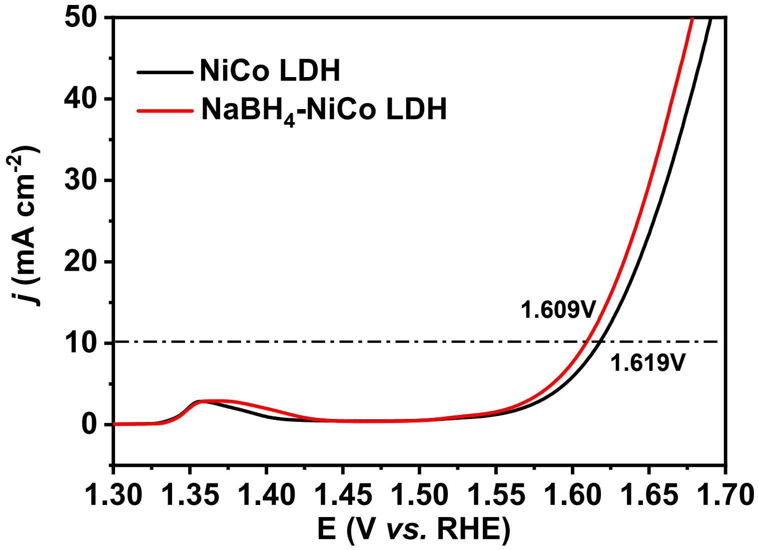

Fig. S16. Linear sweep voltammetry (LSV) curves of the NiCo and $NaBH_4$-NiCo LDHs

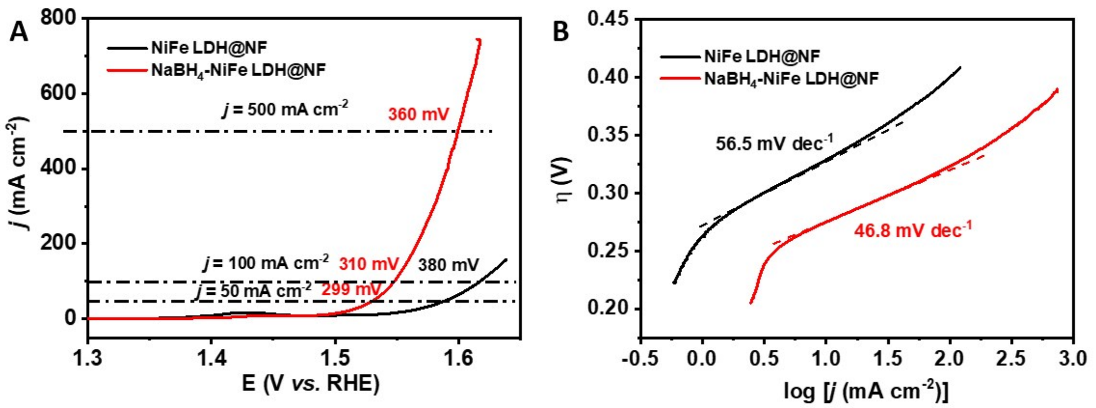

Fig. S17. Electrocatalytic performance characterizations of the catalysts. (A) Linear sweep voltammograms (LSV). (B) Tafel plots.

Table S1. The summarized data from XPS showing the atomic ratios of Ni (III) to Ni (II) and Fe (II) to Fe (III) with various activated time by $NaBH_4$ treatment. $NaBH_4$-NiFe LDH mentioned in the manuscript refers to NiFe LDH treated for 2h, if not otherwise indicated.

Species	Ratio of Ni (III) to Ni (II)	Ratio of Fe (II) to Fe (III)
**NiFe LDH**	0.39	0.40
**1h**	0.78	0.99
**2h**	1.32	1.49
**6h**	0.75	1.51

Table S2. Local structure parameters around Ni estimated by EXAFS analysis.

Sample	Shell	N[a]	R [Å][b]	σ^2 [Å^2][c]	Δ E_0 (eV)	R factor
NiFe-LDH	Ni-O	6.0	2.06	0.0059	-2.1	0.0078
	Ni-Ni	7.6	3.12	0.0087		
	Ni-Fe	1.5	3.06	0.0087		
$NaBH_4$-NiFe-LDH	Ni-O	5.9	2.05	0.0060	-4.4	0.0086
	Ni-Ni	6.8	3.12	0.0096		
	Ni-Fe	1.4	3.06	0.0096		

[a] N = coordination number; [b] R = distance between absorber and backscatter atoms; [c] σ_2= Debye-Waller factor

Table S3. Local structure parameters around Fe estimated by EXAFS analysis.

Sample	Shell	N[a]	R [Å][b]	σ^2 [$Å^2$][c]	ΔE_0 (eV)	R factor
NiFe-LDH	Fe-O	6.0	2.01	0.0047	-1.7	0.0009
	Fe-Ni	7.4	3.10	0.0070		
	Fe-Fe	2.2	2.95	0.0070		
$NaBH_4$-NiFe-LDH	Fe-O	5.6	1.99	0.0062	-0.7	0.0012
	Fe-Ni	5.8	3.09	0.0086		
	Fe-Fe	2.1	2.91	0.0086		

[a] *N* = coordination number; [b] *R* = distance between absorber and backscatter atoms; [c] $\sigma 2$= Debye-Waller factor

Table S4. The summarized data from XPS showing the atomic ratios of Ni (III) to Ni (II) of various $NaBH_4$-NiFe LDH samples before and after CV duration.

Species	Ratio of Ni (III) to Ni (II) (Before)	Ratio of Ni (III) to Ni (II) (After)
**NiFe LDH**	0.39	0.40
**1h**	0.78	0.91
**2h**	1.32	1.50
**6h**	0.75	0.76

Table S5. The summarized data from XPS showing the ratios of M_1/M_2 in LDHs and $NaBH_4$-TM LDHs.

Species	**Mn (II)/Mn (III)**	**Ni (III)/Ni (II)**
**NiMn LDH**	0.79	0.5
**$NaBH_4$-NiMn LDH**	1.53	1.01
	**Co (II)/Co (III)**	**Ni (III)/Ni (II)**
**NiCo**	0.76	0.45
**$NaBH_4$-NiCo**	1.42	1.20
	**Fe(II)/Fe(III)**	**Ni (III)/Ni (II)**
**NiFe**	0.40	0.39
**$NaBH_4$-NiFe**	1.49	1.32

Table S6. Comparison of the catalysts and their OER activity in alkaline medium

**Catalyst**	**Substrate**	**Medium**	**η(mV) for OER @10 mA cm^{-2})**	**η(mV) for OER @20 mA cm^{-2})**	**η(mV) for OER @ 100 mA cm^{-2})**	**η(mV) for OER @ 500 mA cm^{-2})**	**Tafel slope (mV dec^{-1})**	**Ref.**
**3D NiFe LDH/graphene**	Au RDE	1 M KOH	259			-	39	1
**NiFe LDH/carbon quantum dots**	bare GC	1 M KOH	235			-	35	2
**Fe_2O_3@Ni_2P/$Ni(PO_3)_2$**	NF					340		3
**3D NiFe-LDH HMS**			290				51	4
**Au/NiFe LDH**	Ti mesh	1M KOH	237				36	5
**NiFeSe-LDH**	nickel foam	1M KOH	244				32	6
**3D-NiFe-LDH**	nickel foam	0.1M KOH	256				50	7
**NiFe nanoplatelet arrays**	nickel foam	1M KOH	224				52.8	8
**NiFeZn-LDH**	nickel foam	1M KOH		298			63.8	9
**NiFeAl-LDH**	nickel foam	1M KOH		310			69.6	9
**D-NiFeAl-LDH**	nickel foam	1M KOH		270			46.1	9
**NiFe-LDH array**	nickel foam	1 M NaOH	~295				57	10
**Ni_3S_2 nanorod@NiFe LDH nanofilms**	nickel foil	1M KOH	245				35	11
**NiFeMn LDH nanosheets**	Ni Foam	1M KOH		289			47	12
**Our work $NaBH_4$-NiFe LDH@NF**	Ni Foam	1M KOH	250	270	310	368	47	

Au RDE: Au rotating disk electrode

HMS：hollow microsphere
